# Discovery of associative patterns between workplace sound level and physiological wellbeing using wearable devices and empirical Bayes modeling

**DOI:** 10.1038/s41746-022-00727-1

**Published:** 2023-01-13

**Authors:** Karthik Srinivasan, Faiz Currim, Casey M. Lindberg, Javad Razjouyan, Brian Gilligan, Hyoki Lee, Kelli J. Canada, Nicole Goebel, Matthias R. Mehl, Melissa M. Lunden, Judith Heerwagen, Bijan Najafi, Esther M. Sternberg, Kevin Kampschroer, Sudha Ram

**Affiliations:** 1 School of Business, University of Kansas, Lawrence, KS USA; 2 Department of Management Information Systems, Eller College of Management, University of Arizona, Tucson, AZ USA; 3 HKS Inc, Denver, CO USA; 4 Institute on Place, Wellbeing, and Performance, University of Arizona, Tucson, AZ USA; 5 Center for Innovations in Quality, Effectiveness and Safety (IQuESt), Baylor College of Medicine, Houston, TX USA; 6 Department of Medicine, Baylor College of Medicine, Houston, USA; 7 Big Data Scientist Training Enhancement Program, VA Office of Research and Development, Washington, District of Columbia, USA; 8 Office of Federal High Performance Green Buildings, U.S. General Services Administration, Washington, DC USA; 9 Best Buy, Boston, MA USA; 10 Aclima, Inc, San Francisco, CA USA; 11 Department of Psychology, University of Arizona, Tucson, AZ USA; 12 Center to Stream HealthCare in Place (C2SHIP), Michael E. DeBakey Department of Surgery, Baylor College of Medicine, Houston, TX USA; 13 Andrew Weil Center for Integrative Medicine, University of Arizona, Tucson, USA

**Keywords:** Interdisciplinary studies, Neurophysiology, Technology, Statistics

## Abstract

We conducted a field study using multiple wearable devices on 231 federal office workers to assess the impact of the indoor environment on individual wellbeing. Past research has established that the workplace environment is closely tied to an individual’s wellbeing. Since sound is the most-reported environmental factor causing stress and discomfort, we focus on quantifying its association with physiological wellbeing. Physiological wellbeing is represented as a latent variable in an empirical Bayes model with heart rate variability measures—SDNN and normalized-HF as the observed outcomes and with exogenous factors including sound level as inputs. We find that an individual’s physiological wellbeing is optimal when sound level in the workplace is at 50 dBA. At lower (<50dBA) and higher (>50dBA) amplitude ranges, a 10 dBA increase in sound level is related to a 5.4% increase and 1.9% decrease in physiological wellbeing respectively. Age, body-mass-index, high blood pressure, anxiety, and computer use intensive work are person-level factors contributing to heterogeneity in the sound-wellbeing association.

## Introduction

Wellbeing is the ability of the human body to cope with day-to-day stress. On average, four out of ten employees in organizations in the U.S. find their job and workplace stressful and that it adversely affects their health^1^. Past studies have shown that the workplace environment is closely tied to an office worker’s wellbeing markers including mental state, productivity, stress, and longevity^2^. Among environmental stressors, sound level is considered a significant contributor to a variety of adverse health outcomes^3^. The World Health Organization (WHO) has identified elevated sound level or noise as the second leading environmental cause of health problems after air quality, causing serious health effects including stress, coronary heart disease, stroke, and disturbances in communication, rest and sleep^4^. While past research has focused on industrial settings and environmental noise (e.g., aircraft and traffic), research on the effect of more moderate levels of workplace sound on our wellbeing has been lacking due to technological and study design challenges^5^. We conduct a large-scale natural experiment in a real office environment using multiple wearables and develop explainable methods^6^ to meticulously model the sound-wellbeing association. By gaining insights into the association between workplace sound level and physiological wellbeing, organizations can make informed policy changes that impact the longevity, morale, and productivity of its workers.

Our present study enquiring into the sound-wellbeing association is part of the U.S. General Services Administration (GSA)’s Wellbuilt-for-Wellbeing (WB2) program, an interdisciplinary research collaboration^7^ to assess the impact of workplace environment on the wellbeing of white-collar office-workers. Study participants wore two sensors for three days while carrying out their day-to-day activities, a heart and physical activity monitor, and a personal environment quality sensor-based device. Preliminary data analysis using mixed-effects regression models show a significant curvilinear association between sound level and two heart rate variability (HRV) measures – SDNN and normalized-HF. We develop an empirical Bayes model to characterize physiological wellbeing as a function of SDNN and normalized-HF and to quantify its functional relationship with sound level and other predictors. Thereafter, we analyze the heterogeneity in the effect of sound level across study participants using a regularization-based method. We use predictive power assessment to benchmark our methods against alternative methods applicable for tackling the modeling challenges of analyzing multiple outcomes simultaneously and capturing heterogeneity in effects. We show that our proposed methods have better predictive performance than existing methods and are vital to the discovery of associative patterns between workplace sound level and physiological wellbeing. Our study can inform policies affecting the wellbeing of office workers worldwide and contributes to literature in explainable methods for analyzing wearables data.

## Results

### Participant information

A total of 248 office workers expressed interest in participating in our study, representing approximately 12% of the workforce located in areas of the office buildings where recruitment took place. Pregnant women and those wearing pacemakers or insulin pumps were excluded. Participants taking medication known to affect cardiac activity were noted but not excluded. Due to scheduling problems, sickness and exclusionary criteria, 17 office workers did not participate, resulting in a total enrollment of 231 participants. Due to unexpected changes in work schedules, 8 of the 231 participants were only observed for two, rather than the full three days. The participant’s average age was 44.15 (SD = 12.22), 49.78% female, with an average body mass index (BMI) of 27.60 (SD = 6.10).

### Dataset description

Data was collected from participants using an intake survey, a neck-worn environment sensing device, a chest-worn heart and physical activity monitor, and experience sampling mobile surveys recorded every two hours while participants were in the office premises. After pre-processing, our dataset contained 31,557 observations aggregated at five-minute intervals and processed approximately 200,000 min of wearable data streams from the 231 participants. More information about the data and variables can be found in the WB2 program website^7^ and a previous study^8^.

### Pilot analysis

We trained two independent multilevel regression models on our data with SDNN and normalized-HF as respective outcomes. Sound level was included as a fixed effect as well as a random effect in the models. We found that the fixed effect of workplace sound level was significant, both first order as well as second order, in the two models, i.e., $\beta _{Sound,SDNN} = 0.1038$ (*p* < 0.0001, 95% CI = 0.0448–0.1627, Cohen’s d = 0.23), $\beta _{Sound^2,SDNN} = - 0.0075$ (*p* < 0.0001, 95% CI = −0.0096–−0.0054, Cohen’s d = 0.45), $\beta _{Sound,normalized - HF} = - 0.0979$ (*p* < 0.0001, 95% CI = −0.1216 to −0.0742, Cohen’s d = 0.53), and $\beta _{Sound^2,normalized - HF} = 0.0013$ (*p* = 0.015, 95% CI = 0.0003–0.0023, Cohen’s d = 0.17). Furthermore, the quality of fit measured by Akaike Information Criteria (AIC)^9^ for the curvilinear models were better than the corresponding models with only linear effects of sound level. This shows that sound level has a significant curvilinear effect on both physiological wellbeing measures. Secondly, we also found that including sound level as a random effect improves the quality of fit of the models, implying that the association between sound level and physiological wellbeing varies across individuals.

**Fig. 1:**
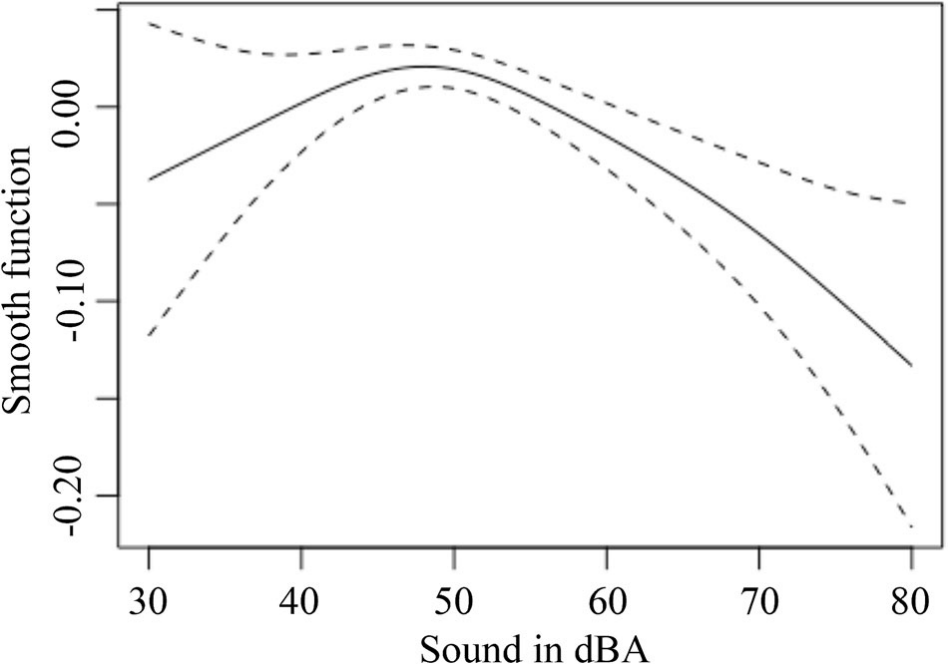
Component smooth function of sound level in GAMM for physiological wellbeing as a bivariate function of SDNN and normalized-HF. The solid line indicates how physiological wellbeing varies as a function of sound level, while the dashed lines are confidence intervals.

The curvilinear association can be further visualized through a smooth function of sound level as a non-parametric input in a Generalized additive mixed model (GAMM)^10^ with outcome as a univariate transformation^11^ of SDNN and normalized-HF. Figure 1 shows the smooth function from GAMM for the sound-wellbeing association having an extremum around 50 dBA. The point estimate of 50 dBA as the optimal sound level was verified using an optimization procedure^12^.

### Population-level sound-wellbeing association

We used an empirical hierarchical Bayes model to simultaneously model the association of sound level with HRV measures – SDNN and normalized-HF, which are common indicators of physiological wellbeing^13–15^. In the model, fixed effects were introduced for inputs: sound level, physical activity level, time of day, day of week, age group, BMI group and gender. Random effects were introduced for sound level and physical activity. We standardized the input (sound level) and outcomes (SDNN and normalized-HF) to remove sensitivity and challenges in posterior estimation convergence due to scale differences in units. The error variances were assigned a diffused half-Cauchy prior, and all other hyperparameters were assigned a diffused Normal prior^16^. The Hamiltonian Monte Carlo algorithm was used for sampling four parallel chains^17^. The R-hat statistic cutoff <1.1 and zero divergence check were used as validation tests for posterior estimates of parameters and assessing quality of fit^17^.

The mean posterior distribution estimates and the 90% credible intervals (between 5th and 95th percentile of the posterior distribution) of the fixed effects coefficients of the empirical Bayes model is given in Table 1. The posterior estimates of the fixed effects indicates a significant association between sound level, time of day, day of week, physical activity level, age, BMI, and physiological wellbeing at workplace.

**Table 1:** Fixed effects of empirical Bayes model.

Coefficients		Posterior estimate (mean)	90% Credible interval
Sound (dBA)	<50	0.0471	(0.0199–0.0648)
	>=50	−0.0167	(−0.0337 to −0.0042)
Physical Activity		0.2756	(0.2316–0.2932)
Time of day	Morning	Baseline	
	Afternoon	−0.1479	(−0.1675 to −0.1277)
	Evening	−0.0939	(−0.1206 to −0.0690)
Day of week	Monday	Baseline	
	Tuesday	−0.1301	(−0.2670–0.0092)
	Wednesday	−0.0571	(−0.0888 to −0.0108)
	Thursday	−0.0588	(−0.0886 to −0.0287)
	Friday	−0.0430	(−0.0836 to –0.0277)
Age (years)	Below 30	Baseline	
	30–40	0.1361	(−0.1439 to –0.4115)
	40–50	−0.1468	(−0.4495 to –0.1641)
	50–60	−0.3119	(−0.6235 to −0.0038)
	Above 60	−0.4413	(−0.7475 to −0.0132)
BMI (kg/m²)	Below 25	Baseline	
	25–30	−0.2278	(−0.4281 to −0.0165)
	30–35	−0.3619	(−0.6751 to −0.0896)
	Above 35	−0.6169	(−0.9768 to −0.2363)
Gender	Male	Baseline	
	Female	−0.0439	(−0.2278–0.1435)

The fixed effect of sound level in the empirical Bayes model represents the sound-wellbeing association on entire study population after accounting for individual heterogeneity as random effects coefficients. The coefficient for sound level indicates a change in physiological wellbeing by a standard deviation (SD) related to a unit standard deviation (SD) change in sound level as both input and outcomes are standardized. Knowing that the SD of sound level in the dataset is 8.79 dBA and the coefficients in Table 1 are standardized, we can compute the unstandardized coefficient estimates to make the following inferences. For sound amplitudes lower than 50 dBA, a 10 dBA increase in sound level is related to a 5.4% (0.95% CI = 2.2–7.4%, Cohen’s d = 0.11) increase in physiological wellbeing. For sound amplitudes higher than 50 dBA, a 10 dBA increase in sound level is related to decrease in physiological wellbeing by 1.9% (0.95% CI = 0.5–3.8%, Cohen’s d = 0.09).

We compared the predictive performance of the empirical Bayes model with the following three alternative methods that can be used for simultaneously modeling two outcomes: (i) a classical univariate transformation method^11^, (ii) a univariate transformation method trained using a Bayesian approach, and (iii) a classical multilevel structural equation modeling method^18^. Models using the classical approach are trained using the R packages lavaan^19^ and nlme^20^ in a 16 GB RAM, 2.7 GHz processor PC, whereas the empirical Bayes model was written and executed using Stan program through the RStan interface^17^, in a high-performance computer cluster with 28 nodes (192 GB RAM per node, Intel Haswell v3 28 core processors). The predictions from the models for SDNN and normalized-HF are compared with the (actual) measured values of the two measures to compute Root Mean Squared Error (RMSE) and Mean Absolute Percentage Error (MAPE)^21^ (Table 2). Table 2 shows that the model trained using the empirical Bayes model has the lowest RMSE and MAPE, indicating that our method is superior to other methods for simultaneous modeling of SDNN and normalized-HF.

**Table 2:** Comparing predictive performance of different simultaneous modeling methods.

Model		SDNN		Normalized-HF	
		RMSE	MAPE	RMSE	MAPE
Classical	Univariate	20.12	34.13	10.98	54.36
	Latent	23.71	44.78	11.22	57.10
Bayesian	Univariate	21.50	37.39	10.04	52.64
	Latent	17.06	26.56	8.90	44.36

Further, we compared the predictive performance of our Bayesian model with five popular machine learning models – Neural Network (NN), Classification And Regression Trees (CART) and Multivariate Adaptive Regression Splines (MARS), Random Forest (RF), and Gradient Boosting Machine (GBM)^21^. We trained these models on training data with and without sound level as an input variable to assess if sound level is a good predictor of SDNN and normalized-HF. The results (Table 3) show that our model outperforms NN, CART, MARS, and its performance is comparable to ensemble learning methods RF and GBM. Except for NN and CART, performance of all other models improves when sound level is included as in input. This shows that sound level is predictive of both the physiological wellbeing measures.

**Table 3:** Comparing predictive performance of machine learning models with and without sound as an input.

Model		SDNN		Normalized-HF	
		RMSE	MAPE	RMSE	MAPE
Without Sound	NN	23.77	45.00	12.56	84.20
	CART	20.58	36.11	11.60	75.43
	MARS	19.85	35.04	11.04	73.57
	RF	17.08	28.78	9.77	57.35
	GBM	17.98	26.55	10.30	51.98
	Our model	17.50	27.80	9.42	50.27
With Sound	NN	23.77	45.00	12.56	84.20
	CART	20.58	36.11	11.60	75.43
	MARS	19.21	33.97	10.83	72.64
	RF	16.96	28.48	9.58	56.79
	GBM	17.05	26.07	9.24	51.35
	Our model	17.06	26.56	8.90	44.36

### Heterogeneity in sound-wellbeing association

**Fig. 2:**
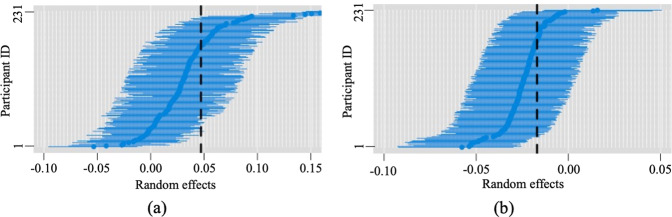
Caterpillar plots of posterior estimates of varying coefficients of sound level and their 60% credible interval in the empirical Bayes model. The vertical dashed line is the fixed effect coefficient while the horizontal blue lines indicate the random effects of sound level on physiological wellbeing across participants. **a** Caterpillar plot for sound level <50 dBA, and **b** the plot for sound level >=50 dBA.

The heterogeneity in sound-wellbeing association across individuals is accounted for by the random effects coefficients of sound level inputs in the empirical Bayes model. Figure 2 shows a caterpillar plot visualization of posterior estimates of random effects of sound level and their 60% credible interval (probability that sample from posteriori distribution falls in given range) in the empirical Bayes model. The vertical lines show the corresponding fixed effects coefficients of sound level. The spread of mean values of posterior estimates of the random effects indicates substantial heterogeneity in the sound-wellbeing association across study participants.

We developed a regularization-based feature-selection method to identify person-level variables contributing to heterogeneity. The person-level variables input into the model were neuroticism, noise sensitivity, age, BMI, presence of high blood pressure (BP), anxiety, sleep problems, computer-use intensive (CUI) worktype, managerial work, meeting intensive work, technical work, and average sound exposure. All person-level variables except age, BMI and average sound exposure were based on a survey completed by the participants at the beginning of the study.

We considered two subsets of the data, one with sound levels <50 dBA (low sound levels) and the other with sound levels ≥50 dBA (high sound levels), to fit two independent sets of models. By fitting two independent sets of models, we were able to make independent inferences about individual heterogeneity effects for each scenario. The coefficients for the regularized feature selection models are in Table 4.

**Table 4:** Coefficients of person-level input variables contributing to heterogeneity.

Predictors		Below 50 dBA			Above 50 dBA		
		Lasso	Elastic-net	Adaptive lasso	Lasso	Elastic-net	Adaptive lasso
Age (years)	Below 30	baseline	baseline	baseline	baseline	baseline	baseline
	30–40				−0.0011	−0.0076	−0.0002
	40–50						
	50–60	−0.0026	−0.0141	−0.0003			
	Above 60	−0.0047	−0.0224	−0.0007	0.0084	0.0160	0.0010
BMI (kg/m²)	Below 25	baseline	baseline	baseline	baseline	baseline	baseline
	25–30	−0.0009	−0.0044	−0.0001		0.0004	
	30–35				−0.0001	−0.0096	
	Above 35				0.0076	0.0123	0.0011
HighBP	Yes	−0.0133	−0.0764	−0.0021	−0.0207	−0.0203	−0.0042
Anxiety	Yes	−0.0015	−0.0013	−0.0002	0.0060	0.0148	0.0007
Computer use intensive work	Yes	0.0187	0.0881	0.0036			

Table 4 shows that Age, BMI, High BP, Anxiety, and CUI work-type are factors contributing to interpersonal variability in the sound-wellbeing association. The blank cells show that coefficients of corresponding variables have been shrunk to zero by the corresponding feature selection method (i.e., lasso, adaptive lasso, elasticnet). For all the person-level variables not listed in Table 4, all three feature selection methods shrunk the corresponding coefficients to zero.

To evaluate the performance of our method, we compared the predictive performance of the empirical Bayes model with three sets of input variables: (i) inputs including no person-level variables as moderators, (ii) inputs including all person-level variables as moderators, and (iii) inputs including person-level variables identified by varying-coefficients modeling method as moderators. Moderators were included as two-way interactions with fixed effect of sound level. Table 5 shows the prediction errors of all three models with respect to SDNN and normalized-HF. The model with specific person-level variables identified using our regularization-based method has the smallest (best) RMSE and MAPE values.

**Table 5:** Performance comparison of models with different set of person-level factors as moderators.

Moderators of sound level in empirical Bayes model	SDNN		Normalized-HF	
	RMSE	MAPE	RMSE	MAPE
No moderators	17.06	26.56	8.90	44.36
All person-level variables	19.66	31.38	11.13	47.73
Person-level variables identified in our study	16.65	24.97	8.41	43.23

**Fig. 3:**
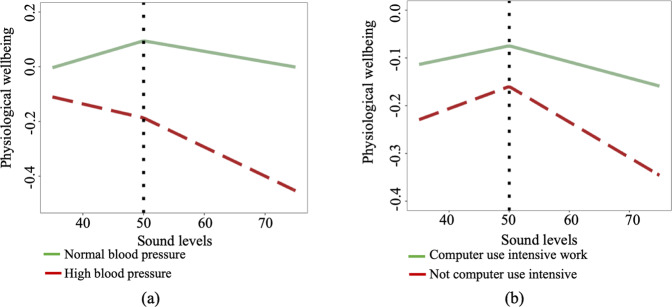
Interaction plots of the top two person-level variables moderating the sound-wellbeing association. **a** Green solid line: Normal blood pressure. Red dashed line: High blood pressure. **b** Green solid line: Computer use intensive work. Red dashed line: Not computer use intensive work.

High BP and Computer-use intensive (CUI) worktype were the person level factors that contributed most to the heterogeneity in sound-wellbeing association. Figure 3(a), (b) are plots showing the change in outcome due to the interaction effects of High BP and CUI worktype variables with the sound level fixed effect in the model. Figure 3(a) shows that office-workers with high blood pressure are more negatively affected than participants with normal blood pressure. Figure 3(b) shows that office-workers involved in CUI work have higher positive effects of sound levels on physiological wellbeing at amplitudes less than 50 dBA, but they have higher negative effects of sound levels on physiological wellbeing at amplitudes over 50 dBA compared to other office-workers.

## Discussion

Workplaces can be designed to evoke positive emotions, stimulate creativity and collaboration, and intensify engagement^22^. On the other hand, unsuitable workplace environments have a potential to cause employee stress and health problems^23^. Psychological wellbeing consists of positive relationships with others, personal mastery, autonomy, a feeling of purpose and meaning in life, and personal growth and development^24^. On the other hand, physiological wellbeing is associated with a dynamic, ever-adapting balance in the human physiological system conditioned by momentary demands^25^.

Sources of sounds in offices include other people’s conversations, telephone-calls, and mechanical equipment. Favorable worker perception of a workplace is tightly coupled with ambient sound level exposure^26–28^. Consequently, sound level is an important workplace environmental factor that could impact employee health and wellbeing^29^. Sound amplitude has been shown to not only affect mood and productivity, but also physiological state of wellbeing^29^. For example, sound levels close to 70 dBA was observed to be optimal for creative cognition^30^, while levels above 85 dBA appeared detrimental to health^31^. In terms of sound-wellbeing association, some studies revealed a negative relationship between high sound levels (i.e., noise) and physiological wellbeing measures^32^, while other studies reported inconclusive results^29,33^. It was also shown that the sources and types of noise do not have a significant effect on physiological wellbeing^34,35^. Also, the effect of sound level on physiological outcomes, if present, were observed to be consistent for low as well as high sound frequencies^32,36^. The nature of the sound-wellbeing relationship has also been observed to be non-monotonic^37^ and instantaneous^38^. A table summarizing studies on sound-wellbeing association has been provided in Supplementary Table 1. Existing studies analyzing the sound-wellbeing association suffer from three major limitations. First, the majority of the studies in the past employed experiments with a limited set of treatments, small sample sizes, and limited number of control variables^29,33,35,36^. Consequently, the results from those studies cannot be easily generalized to the real office workplaces. Second, studies report results from multiple models corresponding to different measures of physiological wellbeing^33–35^ making it difficult to generalize insights and take actions. Third, the sound-wellbeing association has not been precisely quantified using a model^37^. Our present study seeks to address these limitations by conducting a large-scale natural experiment in an office environment using wearables and developing a set of statistical methods to model the sound-wellbeing association.

HRV is the variability between heart beats and is considered as a proxy measure for the physiological wellbeing of a person, i.e., the higher the variability, the higher the wellbeing^15,39^. It is a relatively less intrusive and more reliable measure than recording alternative physiological wellbeing signals such as salivary cortisol and skin conductance^40^. While many measures of HRV exist, each serves as a slightly different lens to view the body’s physiological stress response^41^. The mean of standard deviation for all successive R-R intervals (SDNN) is a global index of HRV and reflects longer term circulation differences or the overall activity in the autonomic nervous system (ANS)^42^. The normalized high frequency component (normalized-HF) of HRV is the ratio between the absolute value of the High Frequency and the difference between Total Power and Very Low Frequency bands in the frequency domain power spectrum of heart rate that emphasizes changes in parasympathetic nervous system (PNS) regulation^42^. SDNN and normalized-HF are indicators of temporal stress and vagal/parasympathetic modulation respectively, and high values of SDNN and normalized-HF have consistently been found to indicate better health and wellbeing^13–15^. Since there is no single unified measure of physiological wellbeing identified in prior literature^15,39–41^, we take an empirical approach by defining physiological wellbeing as a latent (i.e., hidden) variable (*θ*) in a Bayesian model, that captures the variations of SDNN and normalized-HF simultaneously. Other measures of HRV such as RMSSD, SDRR, HF, LF, normalized-LF, LF/HF, Poincare plots^39^ were examined but not considered as outcomes since their variance was either low in our data or their inclusion did not significantly improve the quality of fit of our model.

Existing digital health studies analyzing multiple outcomes fit an independent model for each outcome and report coefficients for each of the models separately^33–35,37^. Interpretation and communication of results from multiple models for decision-making can be challenging. A statistical model with a single set of coefficients for multiple outcomes, known as simultaneous modeling, is suitable for this purpose^11,43,44^. Simultaneous modeling differs from multivariate modeling, where coefficients are estimated for each outcome along with cross-correlation parameters^45,46^. For example, for three outcomes and three inputs, a simultaneous multiple regression model will contain three coefficients (excluding the intercept), whereas a multivariate regression modeling procedure will estimate nine coefficients (excluding the intercepts for outcomes) and corresponding covariance between the coefficients. One approach for simultaneous modeling involves carrying out a univariate transformation of multiple outcomes after accounting for heterogeneity in error variances^11,44,47^. In this univariate transformation method, even though different outcomes have different error variances in the model, the effects of input variables are assumed to be uniform across outcomes. Latent variable modeling is another approach for simultaneous modeling of multiple outcomes^48^. However, classical latent variable modeling approaches such as structural equation modeling requires individual items of the latent construct(s) to be theoretically related and to have construct validity^49^. Moreover, the estimation procedure becomes complex with longitudinal data such as that of wearables^51^. Our proposes an empirical hierarchical Bayesian modeling method to overcome these challenges related to simultaneous modeling of multiple outcomes. While it is useful to understand the population-level effects of input(s) on outcome(s), insights regarding how and why effects differ across individuals can be valuable. The random effects in a multilevel model indicate the presence of individual heterogeneity in input effects^50^. A simple approach to identify factors contributing to individual heterogeneity is to introduce each factor in an interaction term with the input variable and test its significance. This is known as slopes-as-outcomes modeling^50^. However, this approach is sensitive to noise in longitudinal data and becomes cumbersome as the number of potential factors increases^50^ such as in our case. Therefore, we propose the heterogeneity modeling method to identify person-level factors moderating the sound-wellbeing relationship.

Predictive modeling and explanatory modeling go hand in hand as the former predicts the future using existing data, focusing on questions of “What will be”, while the latter illuminates hidden patterns and tells us about “What is” with respect to a phenomenon^51^. Both are important for creating value using data generated from digital sources such as wearables. As wearable technology-based applications increase in the future, the amount of available data to analyze will exponentially increase and warrant more advancements in explanatory modeling for meaningful pattern interpretations. While machine learning methods such as ensemble learners and neural networks can predict outcomes, their ability to explain the functional relationship(s) between input(s) and outcome(s) is limited^21^. Therefore, in this study, we develop new explainable methods for digital data generated from wearables and apply them to explore the sound-wellbeing association. Our study allows researchers and practitioners to not only reconcile some of the differences in past work on the effect of sound on wellbeing, but to also separate out factors that should be controlled for in future work (e.g., blood pressure and nature of work). As wearable technology becomes widely available, personalized measurement is feasible and allows understanding the impact of our surroundings at an individual level. This can improve workplace design, personalized and targeted medicine, and also provide individuals with knowledge to make personal choices to maximize wellbeing. These in turn, improve our ability to function at our best in the workplace.

Our study has the following assumptions and limitations. We focused on modeling the effects of workplace sound levels on the physiological wellbeing of office workers, but we have not collected information about the sound types (e.g., conversation, mechanical background noise, etc.) and frequencies (e.g., low frequency, speech tones, high frequency, etc.) due to individual privacy concerns and sensor technology limitations. However, since prior research has shown that office sound type and frequency do not moderate the effects of sound level on physiological wellbeing outcomes^32,35^, we believe our findings will still hold when controlling for the type and frequency of ambient sounds. Secondly, we have aggregated the sound level and other level-1 variables at 5-min intervals to match the grain of short-term physiological wellbeing HRV measures – SDNN and normalized-HF following clinical guidelines^52,53^. Therefore, the lasting effects of spikes in sound level due to sudden events (e.g., shouting, objects falling or breaking, etc.) or sound level variance within a short timeframe have not been investigated, which can be examined in future research. Nevertheless, the effects of events repeated multiple times as well as background noises consistent across the five-minute interval are accounted for in our models. Next, we hypothesized that SDNN is capable of tracking temporal stress and normalized-HF is capable of tracking vagal/parasympathetic modulation of stress response and their combination is a proxy of physiological wellbeing as both these measures have been shown to be related to physical health and wellness^13–15^. Possible HRVs’ combination both on temporal and spectral domains are an ongoing effort within the research communities. Other combinations of physiological wellbeing indicators can be examined using our method for other scenarios as future research (e.g., LF and HF as physiological wellbeing indicators in a factory setting). Finally, data from each of the 231 participants was collected for a maximum of 3 days, thus our study does not make any inference related to long term effects of sound on physiological wellbeing. Future studies can examine data for a larger study population for a longer period to report long-term effects of workplace sound level on wellbeing.

## Methods

### Study design

The Wellbuilt-for-Wellbeing (WB2)^7^ consisted of a sixteen-month multi-phase field study funded by the U.S. General Services Administration to understand the impact of workplace environment on the wellbeing of white-collar office-workers. In the study, self-described healthy adult workers involved in a variety of office-based roles for the U.S. government were recruited across four federal office buildings across the country. Buildings were selected for their representation of common office workstation types across the U.S. General Services Administration’s portfolio of office space which houses over one million employees. Staff in sections of each office building, from organizations with leadership approval, were offered the opportunity to participate. After giving written informed consent, participants completed an intake survey consisting of demographic questions. Participants wore two sensors for three days while carrying out their day-to-day activities, a heart and physical activity monitor, and a personal environment quality sensor-based device. The study also included experience sampling mobile surveys to collect individuals’ perceived psychological responses at periodic intervals of one to two hours. Our study was approved by the University of Arizona Institutional Review Board.

The HRV measures—SDNN and normalized-HF were calculated using guidelines of the European Society of Cardiology and the North American Society of Pacing and Electrophysiology^52^. Physical activity levels were assessed in *g* (i.e., 1 unit of gravitational force) from the *EcgMove3*’s triaxial accelerometer sensor^54^. Sound levels were aggregated at 5-min intervals to be integrated with physiological wellbeing measures SDNN and normalized-HF, assuming no lagged effects^38^. Only observations with both outcome values present were considered in the analysis. Observations with outcome values above the 99.5th percentile were discarded. Age and BMI were discretized to five and four levels, respectively, for ease of interpretation. Data of participants with less than one hour of recorded data were excluded from analysis. Missing values in input variables were imputed using mean values. Apart from sound level as the input variable and SDNN and normalized-HF as the outcomes, person-level variables (e.g., age group, BMI group, gender, etc.), temporal indicators (time of day, day of the week), and physical activity levels were included as covariates in the statistical models. Observations from day 1 and day 2 of participation of all participants were considered as the training dataset, and day 3 observations were used as the holdout sample (i.e., test dataset) for evaluating the predictive performance of models. The input variables and person-level variables were collected based on prior literature on environment-wellbeing modeling^7,37,55,56^ and domain knowledge. Post stepwise feature selection, only significant inputs were considered in the final model and reported. Summary statistics of the input variables is given in Supplementary Table 2.

### Empirical Bayes model

As mentioned earlier, there is no single theoretical construct that unifies multiple measures of physical wellbeing though there are numerous independent indicators of physiological wellbeing^15,42,57^. SDNN and normalized-HF as HRV measures are differently related to the sympathetic and parasympathetic activities of the autonomous nervous system (ANS)^42^. Instead of analyzing their associations with sound-level separately using two models, an empirical Bayes model makes it possible to combine the two outcomes into a single latent construct of physiological wellbeing which can then be modeled as a function of sound level and other exogenous variables. Following Merkle and Wang (2018)^58^, we define a Bayesian model with a latent variable combining multiple outcomes $Y = \{ y_1,y_2, \ldots ,y_h, \ldots ,y_H\}$ as follows:

1 $$y_h|\theta _i,\gamma _h,\lambda _h,\sigma _{{{\mathrm{h}}}}\sim N\left( {\mu _h,\sigma _h^2} \right)$$

$$\mu _h = \gamma _h + \mathop {\sum}\limits_{k = 1}^m {\lambda _{hk}\theta _k,where\,\theta _k\sim N_m(0,{{{\mathrm{{\Phi}}}}})}$$

In Eq. (1), $N\left( {\mu _h,\sigma _h^2} \right)$ is a normal distribution with a non-informative prior for variance $\sigma _h^2$, $\gamma _h$ is the intercept for outcome *h*, and $\theta _{ik}$ is the $k^{th}$ latent factor value. Φ and $\lambda _{hk}$ are other hyper-parameters to be estimated. In our study, we set $m = 1$ as we have physiological wellbeing as the single latent variable that combines two outcomes SDNN ($y_1$) and normalized-HF ($y_2$). We express the above equation at an observation-level for longitudinal wearables data by adding subscripts *i* and *j* corresponding to the $i^{th}$ observation for the $j^{th}$ individual as shown below:

2 $$y_{ijh}|\theta _{ij},\gamma _h,\lambda _h,\sigma _{{{\mathrm{h}}}}\sim N\left( {\mu _h,\sigma _h^2} \right)$$

$$\mu _h = \gamma _h + \lambda _h\theta _{ij},where\,\theta _{ij}\sim N(0,{{{\mathrm{{\Phi}}}}})$$

The latent variable $\theta _{ij}$ is expressed as an outcome of a mixed-effects model as shown below:

3 $$\theta _{ij} = \beta _0 + \gamma _{0j} + \mathop {\sum}\limits_{k = 1}^K {\beta _kx_{kij}} + \mathop {\sum}\limits_{m = 1}^M {\gamma _{mj}z_{mij} + \xi _{ij}}$$

$$\gamma _{0j}\sim N\left( {0,\sigma _{\gamma _0}^2} \right),\gamma _{mj}\sim N\left( {0,\sigma _{\gamma _m}^2} \right),\xi _{ij}\sim N(0,\sigma _\theta ^2)$$

Upon centering the outcomes and dropping the outcome intercept parameter $\gamma _h$, we can combine the within-individual level error variances (i.e., $\sigma _{ih}^2$ and $\sigma _\theta ^2$). The resultant model is represented as follows:

4 $$y_{hij} - \overline {y_{hij}} = \left( {\beta _0 + \gamma _{0j} + \mathop {\sum}\limits_{k = 1}^K {\beta _kx_{kij}} + \mathop {\sum}\limits_{m = 1}^M {\gamma _{mj}z_{mij}} } \right) \cdot \lambda _h + {\it{\epsilon }}_{ij}^{(h)}$$

$$\gamma _{0j}\sim N\left( {0,\sigma _{\gamma _0}^2} \right),\gamma _{mj}\sim N\left( {0,\sigma _{\gamma _m}^2} \right),{\it{\epsilon }}_{ij}^{(h)}\sim N(0,\sigma _h^2)$$

The empirical Bayes model shown in Eq. (4) can be used for modeling the sound-wellbeing association. The factor loadings, $\lambda _h$, automatically assign different weights to each outcome (i.e., $\lambda _1$ and $\lambda _2$). Alternatively, a latent variable model can be developed using the classical (i.e., frequentist) approach as well, known as a hierarchical Structural Equation Model (SEM). Software such as Mplus, LISREL, EQS, lavaan, OpenMx can fit a two-level SEM with random intercepts^59^. In the two-level SEM model, each outcome $y_{ijh}$ is split into a within and a between component as follows:

5 $$y_{ij} = \left( {y_{ij} - \overline {y_j} } \right) + \overline {y_j} = y_W + y_B$$

In Eq. (5), both the within and between covariance components are treated as orthogonal and additive latent variables^60^. The maximum likelihood estimate for parameters is derived by minimizing the overall loglikelihood which is the sum of likelihood of data from *J* groups. The latent variable model using the classical approach offers less flexibility than its Bayesian counterpart, for it solicits more data-related assumptions and its basic formulation does not account for random effects^60^. Detailed explanations on multilevel modeling using classical statistical modeling and Hierarchical Bayesian modeling is included in the following sub-section.

### Multilevel model inference using classical and Bayesian approaches

Multilevel or hierarchical levels of grouped data are a commonly occurring phenomenon^1^. For example, in organizational studies, information about firms as well as workers are available such that there exists a hierarchical structured data of individual workers nested within multiple firms. Multilevel models (also called as hierarchical linear models, random coefficients models, mixed-effects models) are statistical models with parameters that capture variability across multiple levels of data.

In the classical or frequentist approach, multilevel models can be considered as an extension of an ordinary least squares (OLS) regression model used to analyze variance in the outcome variables when the predictor variables are at varying hierarchical levels. A two-level hierarchical linear model can be mathematically expressed as follows:

6 $${{{\mathrm{Level}}}}\,1:Y_{ij} = \beta _{0j} + \mathop {\sum}\limits_{k = 1}^K {\beta _{kj}V_{kij} + r_{ij}}$$

7 $${{{\mathrm{Level}}}}\,2:\beta _{kj} = \gamma _{k0} + \mathop {\sum}\limits_{m = 1}^M {\gamma _{km}W_{mj} + u_{kj}}$$

In Eq. (6), $Y_{ij}$ is the outcome, $\beta _{kj}$ are the level-1 coefficients, $V_{kij}$ are level-1 input variables, $r_{ij}$ are level-1 residuals, $\gamma _{km}$ are level-2 coefficients, $W_{mj}$ are level-2 input variables and $u_{kj}$ are level-2 variables for $i^{th}$observation of $j^{th}$ individual for $k \in {\Bbb Z}_K$ and $m \in {\Bbb Z}_M$. The assumptions for the model are as follows:

8 $$E\left( {r_{ij}} \right) = 0;var\left( {r_{ij}} \right) = \sigma ^2;E(u_{kj}) = 0;cov\left( {u_{kj},r_{ij}} \right) = 0\forall i,j,k;\left[ {\begin{array}{*{20}{c}} {u_{11}} & \ldots \\ \ldots & {u_{kj}} \end{array}} \right] = T$$

In Eq. (8), *T* is the level-2 variance covariance component that model the inter-relationship between level-2 errors. Combining Eqs. (1) and (2), we can represent hierarchical linear models as follows:

9 $$y_{ij} = \beta _0 + \gamma _{0j} + \mathop {\sum}\limits_{k = 1}^K {\beta _kx_{kij}} + \mathop {\sum}\limits_{m = 1}^M {\gamma _{mj}z_{mij} + {\it{\epsilon }}_{ij}}$$

In Eq. (9), $\beta = \left\{ {\beta _0,\beta _1, \ldots ,\beta _K} \right\}$ are fixed effects coefficients, $\gamma = \left\{ {\gamma _{0j},\gamma _{1j}, \ldots ,\gamma _{Mj}} \right\}$ are random-effects coefficients for *J* groups $j \in {\Bbb Z}_J$, and ${\it{\epsilon }}_{ij}$ is the sum of fixed-effects error and random-effects error components. In matrix notation, the above equation is represented as follows:

10 $$Y = \alpha + X\beta + Z\gamma + {\it{\epsilon }}$$

In Eq. (10), *X* is a matrix of fixed effects and *Z* is a matrix of random effects. Conditional to the above assumptions, the parameters in the model can be estimation by maximizing the likelihood function y as shown below:

11 $$y\sim N(\alpha + X\beta ,\sigma ^2I + Z^\prime TZ)$$

The significance of the fixed effects and random effects are tested using Wald test, Likelihood Ratio Test, F-test, parametric bootstrap or MCMC methods^1^. Model fit can be compared using AIC, deviance and R-squared approximations^2^.

Bayesians, on the other in-hand describe their beliefs about the unknows in a hierarchical linear model before observing data with prior distributions and the following likelihood function:

12 $$y\sim N(\alpha + X\beta + Zb,\sigma ^2I)$$

A single level regression disregards between-group heterogeneity is called model with complete pooling and can yield parameter estimates that are wrong if there is between-group heterogeneity. On the other hand, regression models for each group of the level-2 data independently are called modeling with no pooling and result in imprecise parameter estimates, for they ignore common variance across groups. Hierarchical linear models are considered as a subset of Hierarchical Bayesian models that are models with partial pooling^3^. Parameters are allowed to vary by group at lower levels of the hierarchy while estimating common parameters at higher levels. Note that the level-2 and higher effects are not part of the error variance as in the classical/frequentist approach but modeled as parameters themselves (also called varying coefficients). The varying parameters have hyper-parameters that are estimated based on level-2 and higher order grouping in the data. The estimated posterior distribution of parameters for a hierarchical linear model with normally distributed error and identity link function has the following form:

13 $$p\left( {\alpha ,\beta ,\gamma ,\sigma _Y,\sigma _\gamma \left| {Y,X,Z,U} \right.} \right) \propto$$

$$\mathop {\prod}\limits_{j = 1}^J {\mathop {\prod}\limits_{i = 1}^{n_j} {N\left( {\left. Y \right|\beta _0 + \gamma _{0j} + \beta X + \gamma _jZ,\sigma _Y^2} \right)} } \mathop {\prod}\limits_{j = 1}^J {N\left( {\left. {\gamma _{0j},\gamma _j} \right|\alpha _0 + \alpha U,\sigma _\gamma ^2} \right)}$$

MCMC estimation approaches such as Metropolis Hastings, Gibbs Sampling, and Hamiltonian Monte Carlo families of methods are used to estimate the posterior probability given the prior distribution of all parameters and likelihood of given data^16^. Comparison of implementations and general purpose software packages for classical and Bayesian multilevel modeling is done in West and Galecki^63^, Mai and Zhang^64^, respectively.

### Heterogeneity modeling

As discussed earlier, insights regarding how and why effects differ across individuals can be valuable. We develop a two-step method to find person-level variables explaining the heterogeneity in sound-wellbeing association across individuals. In the first step, we fit an empirical Bayes model with all input variables with random effects coefficients having normal priors with non-zero means. Person-level variables (e.g., age, BMI, gender, etc.) are not included in the model since their value is constant for each individual (i.e., the random effects coefficients for person-level variables have a distribution with zero variance). The empirical Bayes model for step 1 is shown below:

14 $$y_{hij} = \left( {\gamma _{0j} + \mathop {\sum}\limits_{m = 1}^M {\gamma _{mj}z_{mij}} } \right) \cdot \lambda _h + {\it{\epsilon }}_{ij}^{(h)}$$

$$\gamma _{0j}\sim N\left( {\mu _{\gamma _0},\sigma _{\gamma _0}^2} \right),\gamma _{mj}\sim N\left( {\mu _{\gamma _m},\sigma _{\gamma _m}^2} \right),$$

$$m \in {\Bbb Z}_M,{\it{\epsilon }}_{ij}^{(h)}\sim N(0,\sigma _h^2)$$

The mean values $\mu _{\gamma _0}$ and $\mu _{\gamma _m}$ in Eq. (14) are analogous to the model intercept and the corresponding fixed effects coefficients of the $m^{th}$ variable in the empirical Bayesian model shown in Eq. (4). In the second step, we formulate the random effects coefficients of sound level as an outcome of a linear model with person-level variables as the input variables as follows:

15 $$\gamma _{rj} = \beta _0 + \mathop {\sum}\limits_{p = 1}^P {\beta _px_{pj} + {\it{\epsilon }}_j,{\it{\epsilon }}_j\sim N(0,\sigma _r^2)}$$

In Eq. (15), ${{{\mathrm{{\Gamma}}}}}_{{{\mathrm{r}}}} = \left\{ {\gamma _{r1},\gamma _{r2}, \ldots ,\gamma _{rJ}} \right\}$ are the random effects coefficients for the input in the empirical Bayes model from step 1, $\{ x_{1 \cdot },x_{2 \cdot }, \ldots ,x_{P \cdot }\}$ are *P* person-level variables, and ${\it{\epsilon }}_j$ is a normally distributed residual error varying across *J* individuals.

The problem of identifying person-level factors contributing to individual heterogeneity effects is presented as a variable selection problem in our linear model. Traditional stepwise feature selection methods for regression models are ridden with challenges such as sensitivity to changes in data and low external validity^21^. These challenges are particularly relevant in our problem, where there are multiple person-level variables that could be factors contributing to heterogeneity in the sound effects on wellbeing across individuals. Therefore, we choose three regularization-based methods, *lasso*, *elasticnet*, and *adaptive lasso*^21^ to determine significant inputs in the linear model shown in Eq. (15). The *lasso* uses an l-1 penalty to shrink coefficients of insignificant inputs to zero^21^. The *elasticnet* and *adaptive lasso* methods are improvements over the *lasso* feature selection method and account for correlated features and possess oracle properties. The hyperparameters for the penalty functions of these models are determined using a grid-search procedure^21^. The initial adaptive weights are set as inverse of the absolute values of coefficients of a vanilla regression as proposed by Zou^61^. The person-level variables that have non-zero coefficients in all three regularized models are chosen as the final set of factors contributing to individual heterogeneity effects^52^.

**Fig. 4:**
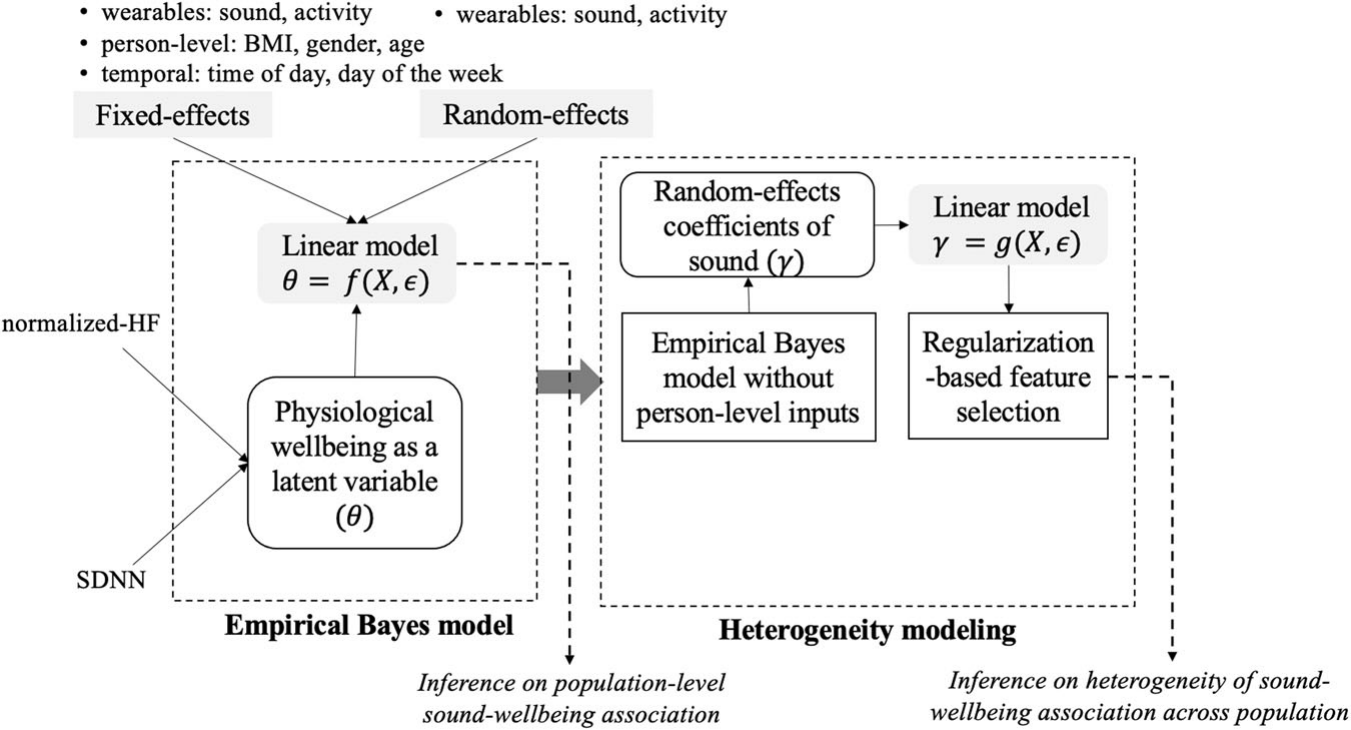
Explanatory modeling framework consisting of an empirical Bayes model and heterogeneity modeling method for identifying population-level and interpersonal sound-wellbeing association.

Figure 4 shows an illustration of our overall explanatory modeling framework consisting of two novel methods for capturing population-level and interpersonal associations between sound levels and physiological wellbeing.

### Experiments

To validate the presence of optimal sound level for physiological wellbeing at 50 dBA and the influence of blood pressure and work involving intensive computer use in moderating the sound-wellbeing relationship, we conducted post-hoc comparison of wellbeing across different stratified populations for three sound level conditions: sound level less than 45 dBA, sound level between 45 dBA and 55 dBA, and sound level greater than 55 dBA. Table 6 shows the post-hoc comparisons of mean wellbeing score adjusted for random effects for the three sound level ranges for different sub-populations in our data. In support of our finding that 50 dBA is an optimal sound level at workplace, we find that sound level range 45–55 dBA has the highest mean adjusted wellbeing score across the complete population, when compared to low and high sound level ranges. However, for individuals with high blood pressure, the lowest sound level range (i.e., sound level < = 45 dBA) is optimal, which is different from individuals with normal blood pressure. Finally, individuals with computer use intensive work have a lower mean adjusted wellbeing score for low as well as high sound level ranges (i.e., sound level <=45 dBA and sound level >55 dBA), when compared to individuals with regular computer use at work. In other words, this group benefits more (than the average individual) from both (a) an increase in sound level at the lower range and (b) a decrease in sound level in the higher range. These post-analysis group comparison findings validate the findings based on our proposed methods.

**Table 6:** Post-hoc group comparisons across sound level ranges.

Sub-population	Mean adjusted wellbeing score^††^		
	Sound <=45 dBA	45 dBA < Sound <=55 dBA	Sound >55 dBA
Complete dataset	0.0054	0.0174	−0.0203
High BP^†^	−0.1395	−0.1600	−0.1884
Normal BP	0.0120	0.0302	−0.0081
Intensive computer use	−0.0407	−0.0186	−0.0985
Regular computer use	0.0229	0.0333	0.0144

^†^Repeated measures MANOVA shows significant differences across sound level ranges for each sub-population except high BP.

^††^Mean value of wellbeing is adjusted for random effects using estimated marginal means procedure^62^.

### Reporting summary

Further information on research design is available in the Nature Research Reporting Summary linked to this article.

## Supplementary information

Related Literature on Sound wellbeing and input variable summary tables

Reporting Summary

## Data Availability

The datasets generated during and/or analyzed during the current study are available from the corresponding author on reasonable request.

## Code availability

The code used for analysis in this study are available from the corresponding author on reasonable request.

## References

[1] Harvard School of Public Health. *The workplace and health*. (2016).

[2] Heerwagen, J. & Zagreus, L. *The human factors of sustainable building design: Post occupancy evaluation of the Philip Merrill Environmental Center*. *Indoor Environmental Quality* (2005).

[3] Daiber, A. et al. Environmental noise induces the release of stress hormones and inflammatory signaling molecules leading to oxidative stress and vascular dysfunction—Signatures of the internal exposome. BioFactors. 45 Preprint at 10.1002/biof.1506 (2019). 10.1002/biof.1506 30937979

[4] WHO. Noise. https://www.euro.who.int/en/health-topics/environment-and-health/noise (2021).

[5] Hicks, J. L. et al. Best practices for analyzing large-scale health data from wearables and smartphone apps. *npj Digital Med.* **2**, (2019). 10.1038/s41746-019-0121-1 PMC6550237 31304391

[6] Loftus ID (2022). Ideal algorithms in healthcare: Explainable, dynamic, precise, autonomous, fair, and reproducible. PLOS Digital Health.

[7] GSA. Wellbuilt for Wellbeing. https://www.gsa.gov/governmentwide-initiatives/federal-highperformance-green-buildings/resource-library/health/wellbuilt-for-wellbeing (2021).

[8] Lindberg CM (2018). Effects of office workstation type on physical activity and stress. Occup. Environ. Med..

[9] Bozdogan H (1987). Model selection and Akaike’s Information Criterion (AIC): The general theory and its analytical extensions. Psychometrika.

[10] Wood, S. N. Stable and efficient multiple smoothing parameter estimation for generalized additive models. *J. Am. Stat. Assoc.* **99**, 673–686 (2004).

[11] Baldwin SA, Imel ZE, Braithwaite SR, Atkins DC (2014). Analyzing multiple outcomes in clinical research using multivariate multilevel models. J. Consulting Clin. Psychol..

[12] Brent, R. P. *Algorithms for minimization without derivatives*. (Dover publications, 2013).

[13] Soares-Miranda L (2014). Physical activity and heart rate variability in older adults. Circulation.

[14] Tan JPH, Beilharz JE, Vollmer-Conna U, Cvejic E (2019). Heart rate variability as a marker of healthy ageing. Int. J. Cardiol..

[15] Natarajan, A., Pantelopoulos, A., Emir-Farinas, H. & Natarajan, P. Heart rate variability with photoplethysmography in 8 million individuals: a cross-sectional study. *The Lancet Digital Health* **2**, e650–e657 (2020). 10.1016/S2589-7500(20)30246-6 33328029

[16] Gelman, A. et al. *Bayesian Data Analysis*. *Bayesian Data Analysis* (CRC Press, 2014).

[17] Carpenter B (2017). Stan: A probabilistic programming language. J. Stat. Softw..

[18] Kline, R. B. *Principles and practice of structural equation modeling*. vol. 156 (Guilford, 2011).

[19] Rosseel Y (2012). lavaan: An R Package for Structural Equation Modeling. J. Stat. Softw..

[20] Pinheiro, J., Bates, D., DebRoy, S. & Sarkar, D. nlme: Linear and Nonlinear Mixed Effects Models. *R package version 3* Preprint at (2007).

[21] Hastie, T., Tibshirani, R. & Friedman, J. *The Elements of Statistical Learning: Data Mining*, *Inference, and Prediction*. (Springer, 2009).

[22] Gruber M, de Leon N, George G, Thompson P (2015). Managing by design. Acad. Manag. J..

[23] Dahl MS (2011). Organizational change and employee stress. Manag. Sci..

[24] Ryff CD (1989). Happiness is everything, or is it? Explorations on the meaning of psychological well-being. J. Personal. Soc. Psychol..

[25] Boron, W. F. & Boulpaep, E. L. *Medical Physiology*. (Elsevier, 2017).

[26] Pitchforth, J., Nelson-White, E., van den Helder, M. & Oosting, W. The work environment pilot: An experiment to determine the optimal office design for a technology company. *PLoS ONE* **15**, e0232943 (2020). 10.1371/journal.pone.0232943 PMC7236992 32428036

[27] Lee, Y., Nelson, E. C., Flynn, M. J. & Jackman, J. S. Exploring soundscaping options for the cognitive environment in an open-plan office. *Building Acoustics* **27**, 185–202 (2020).

[28] Lee PJ, Lee BK, Jeon JY, Zhang M, Kang J (2016). Impact of noise on self-rated job satisfaction and health in open-plan offices: a structural equation modelling approach. Ergonomics.

[29] Jahncke H, Hygge S, Halin N, Green AM, Dimberg K (2011). Open-plan office noise: Cognitive performance and restoration. J. Environ. Psychol..

[30] Mehta R, Zhu R, Cheema A (2012). Is noise always bad? exploring the effects of ambient noise on creative cognition. J. Consum. Res..

[31] OSHA. Occupational Noise Exposure. *Occupational Safety and Health Administration* https://www.osha.gov/noise (2021).

[32] Walker ED, Brammer A, Cherniack MG, Laden F, Cavallari JM (2016). Cardiovascular and stress responses to short-term noise exposures—A panel study in healthy males. Environ. Res..

[33] Cvijanović N, Kechichian P, Janse K, Kohlrausch A (2017). Effects of noise on arousal in a speech communication setting. Speech Commun..

[34] Park SH, Lee PJ (2017). Effects of floor impact noise on psychophysiological responses. Build. Environ..

[35] Sim CS (2015). The effects of different noise types on heart rate variability in men. Yonsei Med. J..

[36] Abbasi, A. M., Motamedzade, M., Aliabadi, M., Golmohammadi, R. & Tapak, L. Study of the physiological and mental health effects caused by exposure to low-frequency noise in a simulated control room. *Building Acoustics* **25**, 233–248 (2018).

[37] Kraus U (2013). Individual daytime noise exposure during routine activities and heart rate variability in adults: a repeated measures study. Environ. Health Perspect..

[38] Srinivasan, K. et al. A Regularization Approach for Identifying Cumulative Lagged Effects in Smart Health Applications. In *Proceedings of the 7th International Conference on Digital Health* 99–103 (ACM Press, 2017).

[39] Gadaleta M (2021). Passive detection of COVID-19 with wearable sensors and explainable machine learning algorithms. npj Digital Med. 2021 4:1.

[40] Verkuil B, Brosschot JF, Tollenaar MS, Lane RD, Thayer JF (2016). Prolonged non-metabolic heart rate variability reduction as a physiological marker of psychological stress in daily life. Ann. Behav. Med..

[41] Xhyheri B, Manfrini O, Mazzolini M, Pizzi C, Bugiardini R (2012). Heart rate variability today. Prog. Cardiovascular Dis..

[42] Shaffer F, Ginsberg JP (2017). An overview of heart rate variability metrics and norms. Front. Public Health.

[43] Das A, Poole WK, Bada HS (2004). A repeated measures approach for simultaneous modeling of multiple neurobehavioral outcomes in newborns exposed to cocaine in utero. Am. J. Epidemiol..

[44] Pituch, K. A. & Stevens, J. P. *Applied Multivariate Statistics for the Social Sciences*. (Routledge, 2016).

[45] Ritz C, Pilmann Laursen R, Trab Damsgaard C (2017). Simultaneous inference for multilevel linear mixed models-with an application to a large-scale school meal study. J. R. Stat. Soc.: Ser. C. (Appl. Stat.).

[46] Lin Y-K, Chen H, Brown RA, Li S-H, Yang H-J (2017). Healthcare predictive analytics for risk profiling in chronic care: A Bayesian multitask learning approach. MIS Q..

[47] Faraway, J. J. *Extending the linear model with R: generalized linear, mixed effects and nonparametric regression models*. (CRC Press, 2016).

[48] Muthén BO (2002). Beyond SEM: General latent variable modeling. Behaviormetrika.

[49] Kline, R. B. *Assumptions in structural equation modeling*. *Handbook of structural equation modeling* (Guilford, 2012).

[50] Raudenbush, S. W. & Bryk, A. S. *Hierarchical Linear Models: Applications and Data Analysis Methods*. (Sage, 2002).

[51] Shmueli G (2010). To explain or to predict?. Stat. Sci..

[52] Malik M (1996). Heart rate variability: Standards of measurement, physiological interpretation, and clinical use. Eur. Heart J..

[53] Pereira T, Almeida PR, Cunha JPS, Aguiar A (2017). Heart rate variability metrics for fine-grained stress level assessment. Computer Methods Prog. Biomedicine.

[54] Razjouyan J (2018). Wearable sensors and the assessment of frailty among vulnerable older adults: an observational cohort study. Sensors.

[55] Thayer JF (2010). Effects of the physical work environment on physiological measures of stress. Eur. J. Cardiovascular Prev. Rehab..

[56] MacNaughton P (2016). Environmental perceptions and health before and after relocation to a green building. Build. Environ..

[57] Richards, D. M. et al. Wearable sensor derived decompensation index for continuous remote monitoring of COVID-19 diagnosed patients. *npj Dig. Med.* **4**, 155 (2021). 10.1038/s41746-021-00527-z PMC8576003 34750499

[58] Merkle EC, Wang T (2018). Bayesian latent variable models for the analysis of experimental psychology data. Psychonomic Bull. Rev..

[59] Skrondal, A. & Rabe-Hesketh, S. *Generalized latent variable modeling: Multilevel, longitudinal, and structural equation models*. (CRC Press, 2004).

[60] Heck, R. H. & Thomas, S. L. *An Introduction to Multilevel Modeling Techniques: MLM and SEM Approaches using Mplus*. (Routledge, 2015).

[61] Zou H (2006). The adaptive lasso and its oracle properties. J. Am. Stat. Assoc..

[62] Searle, S. R., Speed, F. M. & Milliken, G. A. Population marginal means in the linear model: An alternative to least squares means. *Am. Stat. 34,* 216–221 (1980).

[63] West BT, Galecki AT (2011). An Overview of Current Software Procedures for Fitting Linear Mixed Models. Am Stat.

[64] Mai Y, Zhang Z (2018). Software Packages for Bayesian Multilevel Modeling. Struct Equ Modeling.

