# Supplementary material for: Discovery of associative patterns between workplace sound level and physiological wellbeing using wearable devices and empirical Bayes modeling
Source: NPJ Digit Med. 2023 Jan 13;6:5. doi: 10.1038/s41746-022-00727-1 (PMC9839735; doi:10.1038/s41746-022-00727-1)
Supplement: Supplementary file 1 — Related Literature on Sound wellbeing and input variable summary tables [file 41746_2022_727_MOESM1_ESM.pdf]

Supplementary Table 1: Literature on effect of workplace sound levels on physiological wellbeing

Study	Input	Outcome(s)	Study design	Findings
Lusk et al., 2002[1]	Areas with sound levels averaged across a 5 years interval	Blood pressure and heart rate	N=374; Correlating person-level noise exposure with physiological wellbeing; Method: Linear regression	Areas with high sound levels are predictive of increase in blood pressure
Lee et al., 2010[2]	Discrete sound levels	HRV (LF, LF/HF), Mean blood pressure, Mean heart rate	N=16; Treatment = Sound level exposure of No noise, 50 dBA, 60 dBA, 70 dBA and 80 dBA for 5 minutes with 2 minutes interval; Method: Repeated measures ANOVA; Spearman's Rho	HRV decreases with higher sound level exposures, but no change in blood pressure and mean heart rate
Jahncke et al., 2011[3]	Noisy background, river sounds, nature movie	Cortisol, Catecholamines, self-rating of tiredness, mood	N=47; Treatment = Completed tasks for 2 hours each in a low and high noise conditions; Repeated measures ANOVA	Though noisy background and river sounds have an effect on psychological outcomes, they had no significant effect on physiological outcomes
Kraus et al., 2013[4]	Sound levels	HRV (LF/HF, SDNN, RMSSD)	N=110; Prospective panel study with participants spending up to 7.5 hours in a room; Method = Additive mixed models	Sound levels have a positive effect below 65 dBA on SDNN, but is not significantly related to any of the other outcomes
Sim et al., 2015[5]	Sound types, sound levels	HRV (SDNN, HF, LF/HF)	N=40; Treatment: 45 dBA exposure for 5 minutes; Method: Linear regression	Increase in sound level negatively affects physiological wellbeing. Sound types do not have a significant effect on physiological outcomes
Walker et al., 2016[6]	Noise exposure at 75 dBA at low frequency and high-frequency	HRV (SDNN, LF, RMSSD), blood pressure, salivary cortisol, amylase	N=10; Treatment = 40 minutes noise exposure; Method=Multivariate multilevel regression	High sound levels at low-frequencies and high-frequencies have significant negative effect on HRV

Park & Lee, 2017[7]	Floor impact noises ranging from 31.5 dBA to 63 dBA	Noticeability, Annoyance, Heart rate, electrodermal activity, respiration rate	N=21; Treatment = 5 sessions of 15 minutes of different floor impact noises; Method=Repeated measures ANOVA	Annoyance, noticeability, electrodermal activity and respiration rate increases with sound level, but no significant change in heart rate. Physiological responses are not affected by noise source.
Cvijanović et al., 2017[8]	Sound levels	Mental effort, HRV (LF, LF/HF) and skin conductance	N=40; Treatment = 6 dBA background noise added while participants completed collaborative tasks; Method=Multilevel regression	Though mental effort required increases with sound levels, effect on physiological wellbeing was not significant
Srinivasan et al., 2017[9]	Sound levels, Temperature, CO_2, Humidity, Atmospheric pressure	HRV (SDNN, RMSSD, normalized HF, LF/HF)	N=231; Method = Mixed lasso for identify length of cumulative lagged effect of inputs on outcomes	Sound level has an instantaneous effect on HRV whereas other environment factors have a lagged effect of one hour
Abbasi et al., 2018[10]	Low-frequency noise at four different levels	Mental fatigue, LF/HF, working memory	N=35; Method = ANOVA for group comparison in a controlled experiment setup	Mental fatigue caused by low-frequency noise significantly impacted the employees' psycho-physiological and working memory responses

While multiple studies have analyzed the relationship between sound/noise levels in workspace and perceived stress or work performance, fewer studies have examined the physiological implications of workplace sound levels. Table 1 presents a list of studies that have analyzed the relationship between workplace sound levels and physiological wellbeing. The above list does not include studies focusing on psychosocial stress[11], work performance[12], mental wellbeing[13], general workplace environment[14,15], but only studies which have considered

physiological wellbeing as one of their primary outcomes of interest and workplace sound as the input phenomenon.

Supplementary Table 2: Summary statistics of our data

**Variable**	**Summary**			
**INTRAPERSONAL**				
**Numerical**	**Mean**	**SD**	**Units**	**% missing**
SDNN	53.08	23.33	ms	-
Normalized-HF	19.81	12.70	%	-
Sound level	51.85	8.79	dBA	4.29
Physical activity level	0.1738	0.3164	G	0.07
**Categorical**	**Category**	**Hours:Mins**	**Proportion**	**% missing**
Time of day				0.00
	Morning	1224:10	45.76	
	Afternoon	1039:30	38.85	
	Evening	411:15	15.37	
Day of week				0.00
	Monday	449:25	16.80	
	Tuesday	860:50	32.18	
	Wednesday	916:55	34.28	
	Thursday	431:50	16.14	
	Friday	15:45	0.59	
**INTERPERSONAL**				
**Numerical**	**Mean**	**SD**	**Units**	**% missing**
Neuroticism	3.21	0.97	Scale 1-7	10.38
Noise sensitivity	4.05	1.17	Scale 1-7	9.52
Average sound exposure	51.99	4.89	dBA	4.33
**Categorical**	**Category**	**No. of participants**	**Proportion**	**% missing**
Age				9.95
	Less than 30 years	30	12.98	
	30 - 39 years	62	26.83	
	40 - 49 years	43	18.61	
	50 - 59 years	56	24.24	
	60 years or above	17	7.36	
Gender				12.12
	Male	88	38.09	

	Female	115	49.78	
BMI				10.39
	18.5 - 25	76	32.9	
	25.1 - 30	81	35.06	
	30.1 - 35	30	12.98	
	Above 35.1	20	8.66	
Computer-dominant work				8.66
	Yes	93	40.26	
	No	118	51.08	
Management work				8.66
	Yes	69	29.87	
	No	142	61.47	
Technical work				8.66
	Yes	90	38.96	
	No	121	52.38	
Meeting heavy work				8.66
	Yes	42	18.18	
	No	169	73.16	
Sleep problems				9.09
	Yes	42	18.18	
	No	168	72.73	
High blood pressure				9.09
	Yes	42	18.18	
	No	168	72.73	
Anxiety				9.09
	Yes	38	16.45	
	No	172	74.46	

Table 2 shows the summary statistics of relevant intrapersonal variables (i.e., wearable device based repeated measures and temporal information) and interpersonal variables (i.e., person-level information) in this study.

References

1. Lusk, S. L., Hagerty, B. M., Gillespie, B. & Caruso, C. C. Chronic Effects of Workplace Noise on Blood Pressure and Heart Rate. *Archives of Environmental Health* **57**, 273–281 (2002).
2. Lee, G.-S., Chen, M.-L. & Wang, G.-Y. Evoked response of heart rate variability using short-duration white noise. *Autonomic neuroscience : Basic & Clinical* vol. 155 94–97 Preprint at https://doi.org/10.1016/j.autneu.2009.12.008 (2010).
3. Jahncke, H., Hygge, S., Halin, N., Green, A. M. & Dimberg, K. Open-plan office noise: Cognitive performance and restoration. *Journal of Environmental Psychology* **31**, 373–382 (2011).
4. Kraus, U. *et al.* Individual daytime noise exposure during routine activities and heart rate variability in adults: a repeated measures study. *Environ Health Perspect* **121**, 607–612 (2013).
5. Sim, C. S. *et al.* The effects of different noise types on heart rate variability in men. *Yonsei Medical Journal* **56**, 235–243 (2015).
6. Walker, E. D., Brammer, A., Cherniack, M. G., Laden, F. & Cavallari, J. M. Cardiovascular and stress responses to short-term noise exposures—A panel study in healthy males. *Environmental Research* **150**, 391–397 (2016).
7. Park, S. H. & Lee, P. J. Effects of floor impact noise on psychophysiological responses. *Building and Environment* **116**, 173–181 (2017).
8. Cvijanović, N., Kechichian, P., Janse, K. & Kohlrausch, A. Effects of noise on arousal in a speech communication setting. *Speech Communication* **88**, 127–136 (2017).
9. Srinivasan, K. *et al.* A Regularization Approach for Identifying Cumulative Lagged Effects in Smart Health Applications. in *Proceedings of the 7th International Conference on Digital Health* 99–103 (ACM Press, 2017).
10. Abbasi, A. M., Motamedzade, M., Aliabadi, M., Golmohammadi, R. & Tapak, L. Study of the physiological and mental health effects caused by exposure to low-frequency noise in a simulated control room. *Building Acoustics* **25**, (2018).
11. Leather, P., Beale, D. & Sullivan, L. Noise, psychosocial stress and their interaction in the workplace. *Journal of Environmental Psychology* **23**, (2003).
12. Nielsen, K. *et al.* Workplace resources to improve both employee well-being and performance: A systematic review and meta-analysis. *Work and Stress* Preprint at https://doi.org/10.1080/02678373.2017.1304463 (2017).
13. Goh, J., Pfeffer, J. & Zenios, S. A. The relationship between workplace stressors and mortality and health costs in the United State. *Management Science* (2016).
14. Thayer, J. F. *et al.* Effects of the physical work environment on physiological measures of stress. *European journal of cardiovascular prevention and rehabilitation* **17**, 431–9 (2010).
15. Colenberg, S., Jylhä, T. & Arkesteijn, M. The relationship between interior office space and employee health and well-being–a literature review. *Building Research and Information* **49**, (2021).
