# Supplementary material for: Discovery of associative patterns between workplace sound level and physiological wellbeing using wearable devices and empirical Bayes modeling
Source: NPJ Digit Med. 2023 Jan 13;6:5. doi: 10.1038/s41746-022-00727-1 (PMC9839735; doi:10.1038/s41746-022-00727-1)
Supplement: Supplementary file 2 — Reporting Summary [file 41746_2022_727_MOESM2_ESM.pdf]

nature portfolio

Corresponding author(s): Karthik Srinivasan NPJDIGITALMED-03614R
Last updated by author(s): Aug 16, 2022

Reporting Summary

Nature Portfolio wishes to improve the reproducibility of the work that we publish. This form provides structure for consistency and transparency in reporting. For further information on Nature Portfolio policies, see our Editorial Policies and the Editorial Policy Checklist.

Statistics

For all statistical analyses, confirm that the following items are present in the figure legend, table legend, main text, or Methods section.

n/a	Confirmed	
☐	☒	The exact sample size (*n*) for each experimental group/condition, given as a discrete number and unit of measurement
☐	☒	A statement on whether measurements were taken from distinct samples or whether the same sample was measured repeatedly
☐	☒	The statistical test(s) used AND whether they are one- or two-sided<br>*Only common tests should be described solely by name; describe more complex techniques in the Methods section.*
☐	☒	A description of all covariates tested
☐	☒	A description of any assumptions or corrections, such as tests of normality and adjustment for multiple comparisons
☐	☒	A full description of the statistical parameters including central tendency (e.g. means) or other basic estimates (e.g. regression coefficient) AND variation (e.g. standard deviation) or associated estimates of uncertainty (e.g. confidence intervals)
☐	☒	For null hypothesis testing, the test statistic (e.g. *F*, *t*, *r*) with confidence intervals, effect sizes, degrees of freedom and *P* value noted<br>*Give P values as exact values whenever suitable.*
☐	☒	For Bayesian analysis, information on the choice of priors and Markov chain Monte Carlo settings
☐	☒	For hierarchical and complex designs, identification of the appropriate level for tests and full reporting of outcomes
☐	☒	Estimates of effect sizes (e.g. Cohen's *d*, Pearson's *r*), indicating how they were calculated

Our web collection on statistics for biologists contains articles on many of the points above.

Software and code

Policy information about availability of computer code

Data collection	Zephyr, Aclima wearable sensor software
Data analysis	R programming language, Stan programming language for Bayesian analysis

For manuscripts utilizing custom algorithms or software that are central to the research but not yet described in published literature, software must be made available to editors and reviewers. We strongly encourage code deposition in a community repository (e.g. GitHub). See the Nature Portfolio guidelines for submitting code & software for further information.

Data

Policy information about availability of data

All manuscripts must include a data availability statement. This statement should provide the following information, where applicable:

- Accession codes, unique identifiers, or web links for publicly available datasets
- A description of any restrictions on data availability
- For clinical datasets or third party data, please ensure that the statement adheres to our policy

The datasets generated during and/or analyzed during the current study are available from the corresponding author on reasonable request.

Human research participants

Policy information about studies involving human research participants and Sex and Gender in Research.

Reporting on sex and gender	49.78% of participants identified as female gender while the rest were male.
Population characteristics	Average age of participant was 44.15 years (SD=12.22) and BMI was 27.60 (SD = 6.10).
Recruitment	A total of 248 office workers expressed interest in participating in our study, representing approximately 12% of the workforce located in areas of the office buildings where recruitment took place. Pregnant women and those wearing pacemakers or insulin pumps were excluded. Participants taking medication known to affect cardiac activity were noted but not excluded. Due to scheduling problems, sickness and exclusionary criteria, 17 office workers did not participate, resulting in a total enrollment of 231 participants.
Ethics oversight	Our study was approved by the University of Arizona Institutional Review Board.

Note that full information on the approval of the study protocol must also be provided in the manuscript.

Field-specific reporting

Please select the one below that is the best fit for your research. If you are not sure, read the appropriate sections before making your selection.

☐ Life sciences ☒ Behavioural & social sciences ☐ Ecological, evolutionary & environmental sciences

For a reference copy of the document with all sections, see nature.com/documents/nr-reporting-summary-flat.pdf

Behavioural & social sciences study design

All studies must disclose on these points even when the disclosure is negative.

Study description	We conducted a field study using multiple wearable devices on 231 federal office workers to assess the impact of the indoor environment on individual wellbeing.
Research sample	A total of 248 office workers expressed interest in participating in our study, representing approximately 12% of the workforce located in areas of the office buildings where recruitment took place. Pregnant women and those wearing pacemakers or insulin pumps were excluded. Participants taking medication known to affect cardiac activity were noted but not excluded. Due to scheduling problems, sickness and exclusionary criteria, 17 office workers did not participate, resulting in a total enrollment of 231 participants. Due to unexpected changes in work schedules, 8 of the 231 participants were only observed for two, rather than the full three days. The participant's average age was 44.15 (SD=12.22), 49.78% female, with an average body mass index (BMI) of 27.60 (SD = 6.10).
Sampling strategy	Participants were recruited using convenience sampling, in which office workers from 3 office buildings enrolled out of personal interest.
Data collection	Data was collected from participants using an intake survey, a neck-worn environment sensing device, a chest-worn heart and physical activity monitor, and experience sampling mobile surveys recorded every two hours while participants were in the office premises. After pre-processing, our dataset contained 31,557 observations aggregated at five-minute intervals and processed approximately 200,000 minutes of wearable data streams from the 231 participants.
Timing	The field study was conducted in multiple phases/cohorts during May 2015 and August 2016. Within each cohort, participants were expected the health monitors and environment sensing devices for three days.
Data exclusions	Pregnant women and those wearing pacemakers or insulin pumps were excluded.
Non-participation	Due to scheduling problems, sickness and exclusionary criteria, 17 office workers did not participate.
Randomization	Allocation was not random, covariates related to short-term physiological wellbeing were measured and controlled for.

Reporting for specific materials, systems and methods

We require information from authors about some types of materials, experimental systems and methods used in many studies. Here, indicate whether each material, system or method listed is relevant to your study. If you are not sure if a list item applies to your research, read the appropriate section before selecting a response.

Materials & experimental systems

n/a	Involved in the study	
☒	☐	Antibodies
☒	☐	Eukaryotic cell lines
☒	☐	Palaeontology and archaeology
☒	☐	Animals and other organisms
☒	☐	Clinical data
☒	☐	Dual use research of concern

Methods

n/a	Involved in the study	
☒	☐	ChIP-seq
☒	☐	Flow cytometry
☒	☐	MRI-based neuroimaging
